# Comparison of prostate volume measured by transabdominal ultrasound and MRI with the radical prostatectomy specimen volume: a retrospective observational study

**DOI:** 10.1186/s12894-023-01234-5

**Published:** 2023-04-17

**Authors:** Shikuan Guo, Jingliang Zhang, Jianhua Jiao, Zeyu Li, Peng Wu, Yuming Jing, Weijun Qin, Fuli Wang, Shuaijun Ma

**Affiliations:** Department of Urology, Xijing Hospital, Fourth Military Medical University, Xi’an, 710032 China

**Keywords:** Prostate cancer, Magnetic resonance imaging, Prostate volume, Transabdominal ultrasound

## Abstract

**Background:**

Few studies have compared the use of transabdominal ultrasound (TAUS) and magnetic resonance imaging (MRI) to measure prostate volume (PV). In this study, we evaluate the accuracy and reliability of PV measured by TAUS and MRI.

**Methods:**

A total of 106 patients who underwent TAUS and MRI prior to radical prostatectomy were retrospectively analyzed. The TAUS-based and MRI-based PV were calculated using the ellipsoid formula. The specimen volume measured by the water-displacement method was used as a reference standard. Correlation analysis and intraclass correlation coefficients (ICC) were performed to compare different measurement methods and Bland Altman plots were drawn to assess the agreement.

**Results:**

There was a high degree of correlation and agreement between the specimen volume and PV measured with TAUS (r = 0.838, p < 0.01; ICC = 0.83) and MRI (r = 0.914, p < 0.01; ICC = 0.90). TAUS overestimated specimen volume by 2.4ml, but the difference was independent of specimen volume (p = 0.19). MRI underestimated specimen volume by 1.7ml, the direction and magnitude of the difference varied with specimen volume (p < 0.01). The percentage error of PV measured by TAUS and MRI was within ± 20% in 65/106(61%) and 87/106(82%), respectively. In patients with PV greater than 50 ml, MRI volume still correlated strongly with specimen volume (r = 0.837, p < 0.01), while TAUS volume showed only moderate correlation with specimen (r = 0.665, p < 0.01) or MRI volume (r = 0.678, p < 0.01).

**Conclusions:**

This study demonstrated that PV measured by MRI and TAUS is highly correlated and reliable with the specimen volume. MRI might be a more appropriate choice for measuring the large prostate.

**Supplementary Information:**

The online version contains supplementary material available at 10.1186/s12894-023-01234-5.

## Background

Prostate volume (PV) has been proved useful in prostate cancer (PCa) screening and risk stratification. Various clinical risk-stratification nomograms incorporated PV as an important factor, which facilitated the selection of the most appropriate treatment regimen, reduced overdiagnosis and over-treatment of clinically insignificant PCa, and predicted lateral-specific extracapsular extension, lymph node invasion, biochemical recurrence, and clinical recurrence [1]. Prostate specific antigen density calculated by PV and prostate specific antigen (PSA) had a higher predictive value than PSA alone, both for overall and clinically significant PCa [2, 3]. PV was also negatively associated with tumor detection rate at biopsy [4]. Cancer detected in smaller prostate glands was more aggressive, with a higher incidence of advanced carcinoma, extracapsular invasion and seminal vesicle invasion after biopsy and radical resection [5]. In addition, a larger prostate had a significant negative impact on early and late continence after radical prostatectomy (RP)[6].

PV can be measured using a variety of imaging techniques, including ultrasound, computed tomography (CT), or magnetic resonance imaging (MRI). Transrectal ultrasound (TRUS) using ellipsoid formula has long been the preferred imaging modality and has been shown to be comparable to excised cadaveric weights [7]. Still, it was an invasive procedure that could cause discomfort and anxiety. In contrast, transabdominal ultrasound (TAUS) has also been widely used to measure prostate dimensions in the clinic. CT has been frequently used for PV measurement in dose planning and target definition for both external beam radiotherapy and brachytherapy [8]. In addition, MRI has been demonstrated to have adequate diagnostic accuracy in detecting PCa and is widely used as a non-invasive test for PV assessment.

In the development of a nomogram for PCa risk stratification, different measurements lead to differences in PV, which would degrade the performance of the nomogram. To the best of our knowledge, the comparison of TAUS-based or MRI-based volume with actual PV has not been well established. Therefore, the aim of this study was to evaluate the accuracy and reliability of TAUS compared to MRI for estimating prostate volume.

**Methods**.

### Study population

The retrospective study was conducted in a large tertiary care hospital in China. From January 2021 to August 2022, 159 patients diagnosed with prostate cancer underwent laparoscopic or robot-assisted radical prostatectomy. Patients who had both TAUS and MRI examinations within 3 months prior to RP were included. Patients who had previously received transurethral prostate resection, androgen deprivation therapy, or radiotherapy were excluded. Finally, a total of 106 patients were included in the analysis.

### TAUS volume measurement

**Fig. 1:**
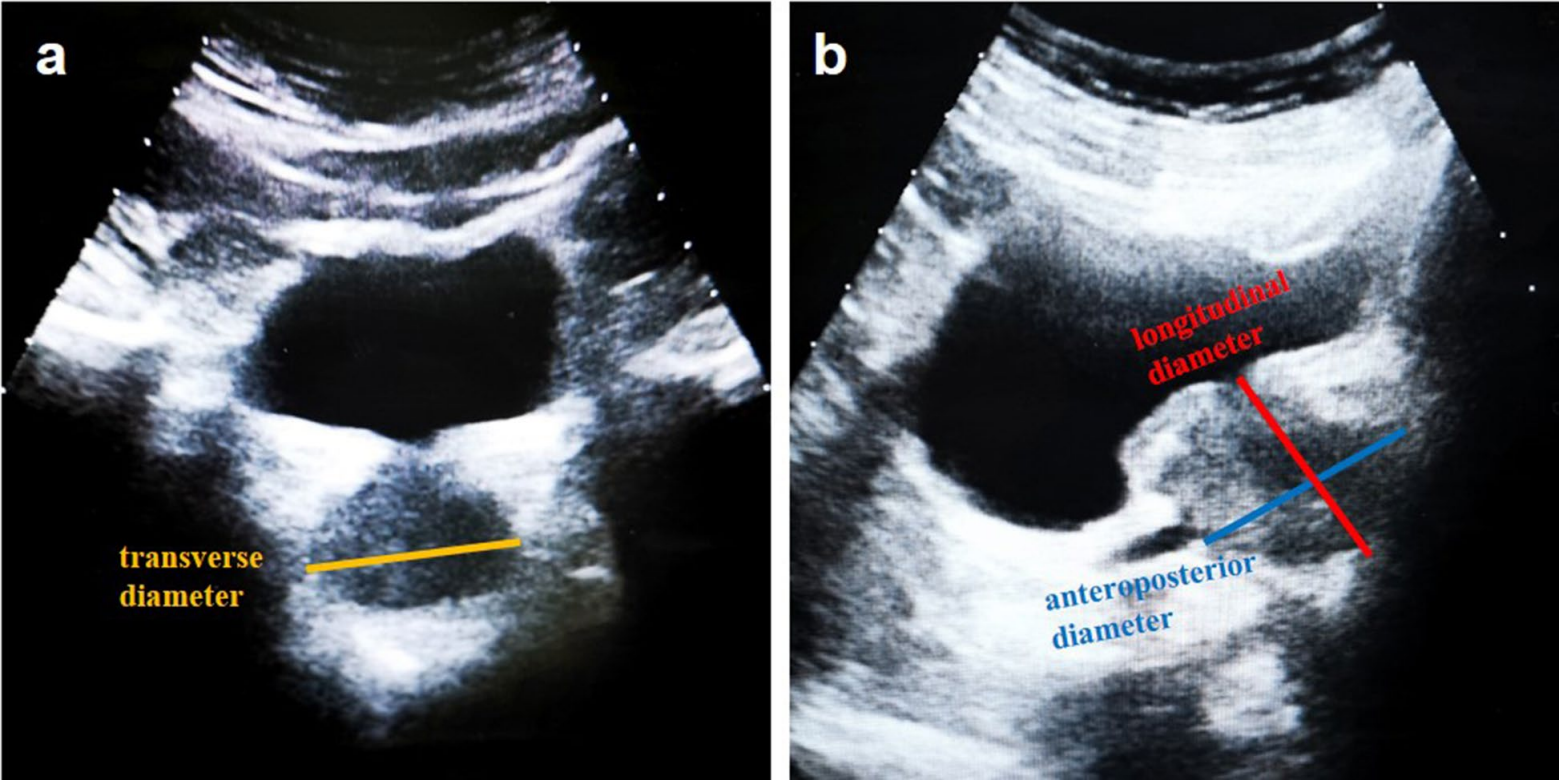
Measurement of prostate diameter when using ellipsoid formula to calculate prostate volume on transabdominal ultrasound. (**a**) maximum transverse diameter (width) measured on axial scanning. (**b**) Maximum longitudinal diameter (length) and maximum anteroposterior diameter (height) measured on midsagittal scanning

Ultrasonographic examinations were performed using a MyLab Twice ultrasound system (Esaote, Italy) with a 3.5 MHz convex probe (CA541, Esaote). Prostatic dimensions on TAUS were extracted from the most recent sonography reports prior to surgery. The patient underwent TAUS in the supine position with a full bladder, which was determined as the patient having a desire to micturate, but no severe discomfort. The ultrasound results were interpreted by two experienced sonographers (with 15 years’ experience and 10 years’ experience) and any discrepancy was discussed to consensus. Representative images of the prostate diameter measurement on TAUS were shown in Fig. 1 and PV was calculated using the ellipsoid formula ($volume=width \times height \times lenght\times \pi /6$).

### MRI volume measurement

**Fig. 2:**
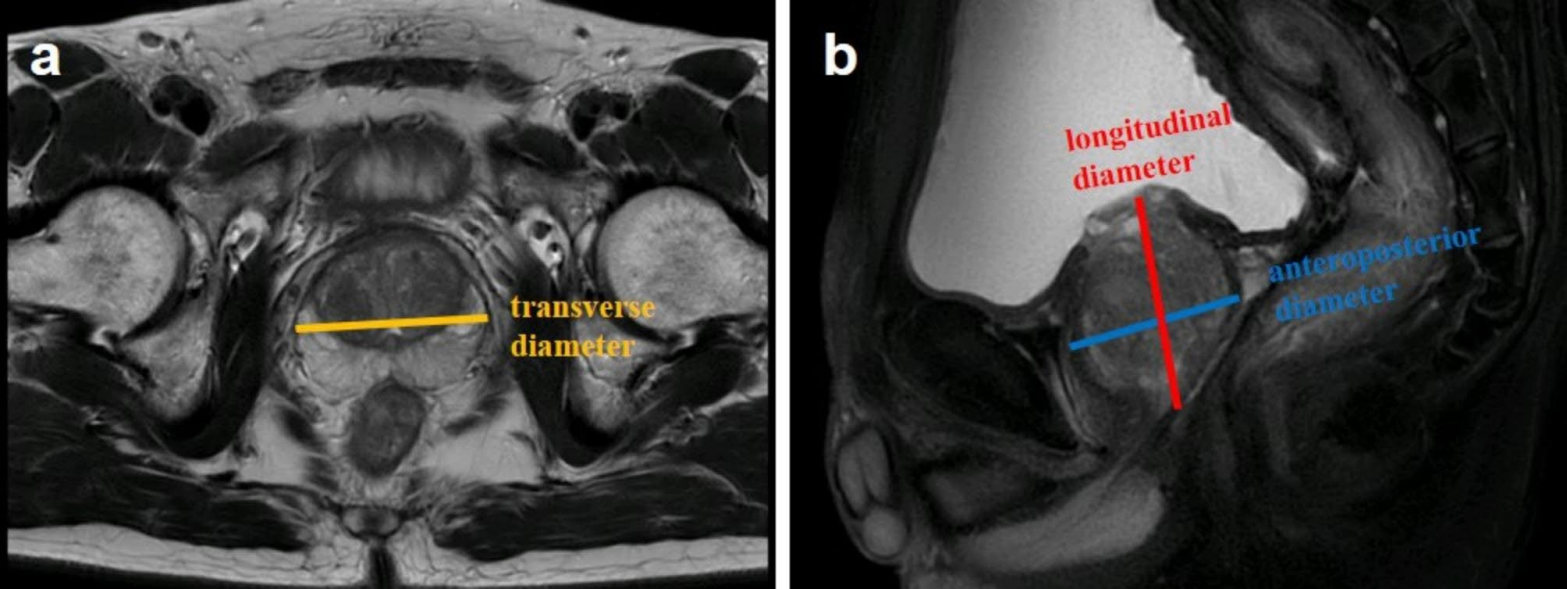
Measurement of prostate diameter when using ellipsoid formula to calculate prostate volume on MRI. (**a**) maximum transverse diameter (width) measured on axial T2W MRI. (**b**) Maximum longitudinal diameter (length) and maximum anteroposterior diameter (height) measured on midsagittal T2W MRI. T2W = T2 weighted; MRI = magnetic resonance imaging

MRI was performed on a 3.0-T superconducting unit (Magnetom Trio, Siemens, Erlangen, Germany). In MRI, the prostate was examined from the apex to base. The maximum transverse (width) diameter was measured on axial T2-weighted images and the maximum longitudinal (length) and anteroposterior (height) diameter were measured on mid-sagittal T2-weighted images (Fig. 2), which was according to version 2.1 of the prostate imaging reporting and data system (PI-RADS 2.1) [9]. All MRI images were blinded reviewed by two experienced radiologists with 8 and 10 years of experience. Any discrepancy will be resolved in consensus with a third radiologist (with 15 years of experience). The MRI-based PV was calculated using the ellipsoid formula ($volume=width \times height\times length \times \pi /6$).

### Specimen volume measurement

The specimen volume was measured using water displacement method in the operating room instantly after radical prostatectomy. After removal of the periprostatic fat, seminal vesicles and vas deferens, the specimen was then immersed in a graduated cylinder of 100ml or 200ml filled with distilled water. The volume of the displaced water was considered equal to the actual PV.

### Statistics analysis

Descriptive statistics were presented as the mean (standard deviation) or median (quartile) for continuous variables, while categorical variables were presented as frequencies and percentages. Paired t-test was utilized to compare PV measured by TAUS and MRI to specimen volume. The percentage error was expressed as the percentage of each difference to its matched reference value. Accuracy was assessed by calculating the mean absolute percentage of error (MAPE) for each method. A MAPE below 20% was considered accurate in this study. The correlation was calculated using Pearson bivariate correlation. Linear regression analysis was used to calculate the percentage of variability in the PV measured with TAUS and MRI, and a linear regression equation was developed. Inter-rater agreement in the different types of volume measurements and specimen volume were assessed by interclass correlation coefficients (ICC). Bland–Altman plots were created, along with the mean difference and 95% limit of agreement (LOA), to show the relationship between the different types of volume measurements and specimen volume. Statistical significance was defined as α < 0.05, and all tests performed were two-tailed. All figures were created using GraphPad Prism (version 9.4.1). All statistical analyzes were performed using IBM SPSS (version 26.0; IBM Corp, Armonk, NY, USA).

## Results

According to the inclusion and exclusion criteria, the final study cohort consisted of 106 patients. The overall demographic and clinical characteristics were shown in Table 1.

**Table 1:** Baseline demographics and clinical characteristics of enrolled patients

Variable	Value
number of patients	106
Age, years	67 (61–73)
PSA, ng/ml	11.48 (7.73–17.45)
TAUS PV, ml	48.5 ± 20.2
MRI PV, ml	44.4 ± 15.4
Specimen volume, ml	46.1 ± 18.3
Pathological T stage	
T2a	11 (10.4)
T2b	13 (12.3)
T2c	56 (52.8)
T3a	21 (19.8)
T3b	5 (4.7)
Surgical Gleason grade group	
1 (3 + 3)	8 (7.5)
2 (3 + 4)	22 (20.8)
3 (4 + 3)	27 (25.5)
4 (8)	35 (33.0)
5 (9–10)	14 (13.2)

Values were presented as mean ± standard deviation, number (%), or median (interquartile range). MRI, magnetic resonance imaging; PSA, prostate specific antigen; PV, prostate volume; SD, standard deviation; TAUS, transabdominal ultrasound.

### Comparison between TAUS volume and specimen volume

**Fig. 3:**
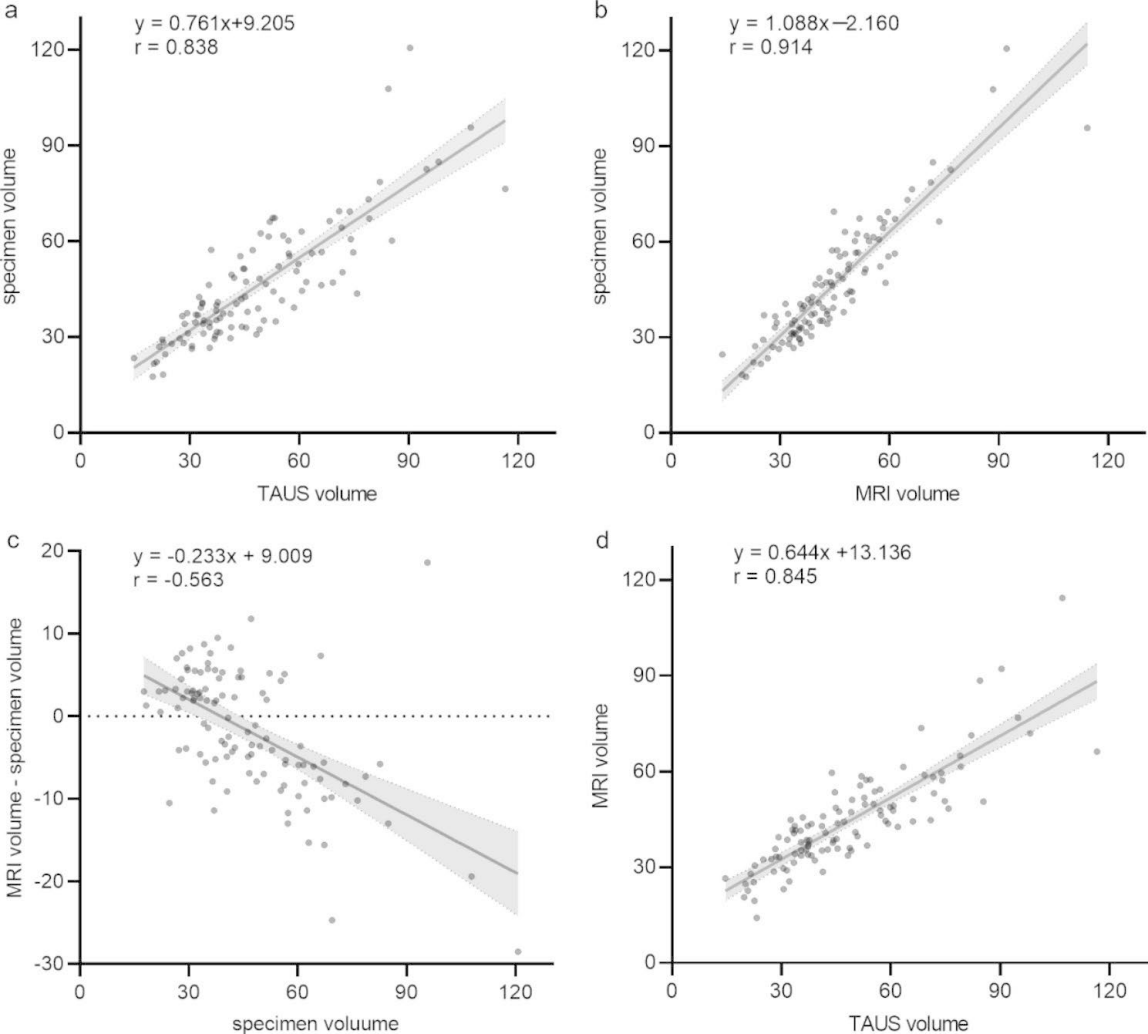
Scatterplot examination and linear regression analysis between different prostate volume measurements. (**a**) TAUS volume compared with the specimen volume; (**b**) MRI volume compared with the specimen volume; (**c**) the difference between MRI volume and specimen volume compared with the specimen volume; (**d**) TAUS volume compared with MRI volume. MRI, magnetic resonance imaging; TAUS, transabdominal ultrasound

There was a strong correlation between TAUS volume and specimen volume (r = 0.838, P < 0.01) (Fig. 3a). Paired t-test revealed a statistically significant difference (P < 0.05) and TAUS overestimated specimen volume by 2.4ml on average. The volume-dependent relationship between the difference and specimen volume was not observed (P = 0.19).

**Fig. 4:**
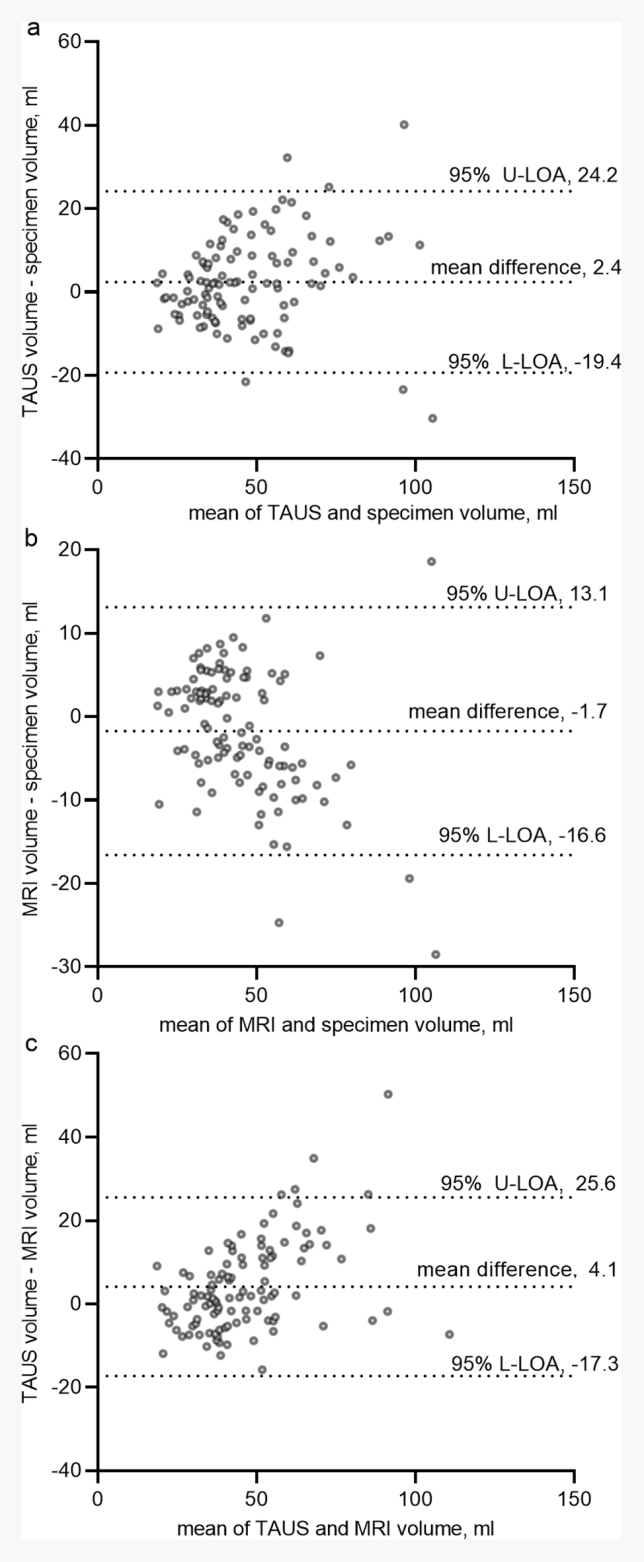
Bland-Altman plots show comparisons between (**a**) TAUS volume and the specimen volume; (**b**) MRI volume and the specimen volume; (**c**) TAUS volume and MRI volume. U-LOA, upper limit of agreement; L-LOA, lower limit of agreement; MRI, magnetic resonance imaging; TAUS, transabdominal ultrasound

The ICC for TAUS volume and specimen volume was 0.83 (95% CI, 0.77‒0.88), indicating good reliability. The Bland-Altman plots representing the relationship between the difference and the means of TAUS volume and specimen volume was shown in Fig. 4a. The MAPE of TAUS in estimating prostate size was 18.9%. For the entire cohort, the number of people with percentage error within ± l0%, ± 20%, and ± 30% were 34/106 (32%), 65/106 (61%), and 85/106 (80%), respectively (Table 2).

**Table 2:** Accuracy of PV measured by different methods

Variable	Percentage error within			MAPE
	± 10%	± 20%	± 30%	
TAUS PV vs. specimen volume	34 (32%)	65 (61%)	85 (80%)	18.9%
MRI PV vs. specimen volume	45 (42%)	87 (82%)	103(97%)	13.2%
TAUS PV vs. MRI PV	36 (34%)	60 (57%)	88 (83%)	19.6%

Values were presented as number of patients (%) or percentage; MRI, magnetic resonance imaging; MAPE, mean absolute percentage of error; PV, prostate volume; TAUS, transabdominal ultrasound.

### Comparison between MRI volume and specimen volume

There was a strong correlation between MRI volume and specimen volume (r = 0.941, P < 0.01) (Fig. 3b). Paired t-test showed a statistically significant difference (P < 0.05) and MRI underestimated specimen volume by 1.7ml on average. Linear regression analysis showed that the difference was negatively related to specimen volume (r=-0.563, p < 0.01). The direction and magnitude of the difference varied with the specimen volume. If specimen volume was < 39 ml, MRI overestimated the specimen volume; if specimen volume was > 39 ml, MRI underestimated the specimen volume (Fig. 3c).

The ICC was 0.90 (95% CI, 0.86‒0.93), providing very strong reliability. The Bland-Altman plots representing the relationship between the difference and means of MRI volume and specimen volume was shown in Fig. 4b. The MAPE of MRI in estimating the specimen volume was 13.2%. For the entire cohort, the number of people with percentage error within ± l0%, ± 20%, and ± 30% were 45/106 (42%), 87/106 (82%), and 103/106 (97%), respectively (Table 2).

### Comparison between TAUS volume and MRI volume

In addition, we also analyzed the correlation between PV measured by TAUS and MRI (r = 0.845, p < 0.01) (Fig. 3d). Paired t-test indicated a statistically significant difference between PV measured by TAUS and MRI (P < 0.01) and TAUS overestimated MRI volume by 4.1ml on average.

The ICC was 0.82 (95% CI, 0.74‒0.87), providing a good reliability. The Bland-Altman plots depicting the relationship between the difference and means of TAUS volume and MRI volume was shown in Fig. 4c. The MAPE was 19.6%. For the entire cohort, the number of people with percentage error within ± l0%, ± 20%, and ± 30% were 36/106 (34%), 60/106 (57%), and 88/106 (83%), respectively (Table 2).

### Comparison in patients with PV bigger than 50ml

**Fig. 5:**
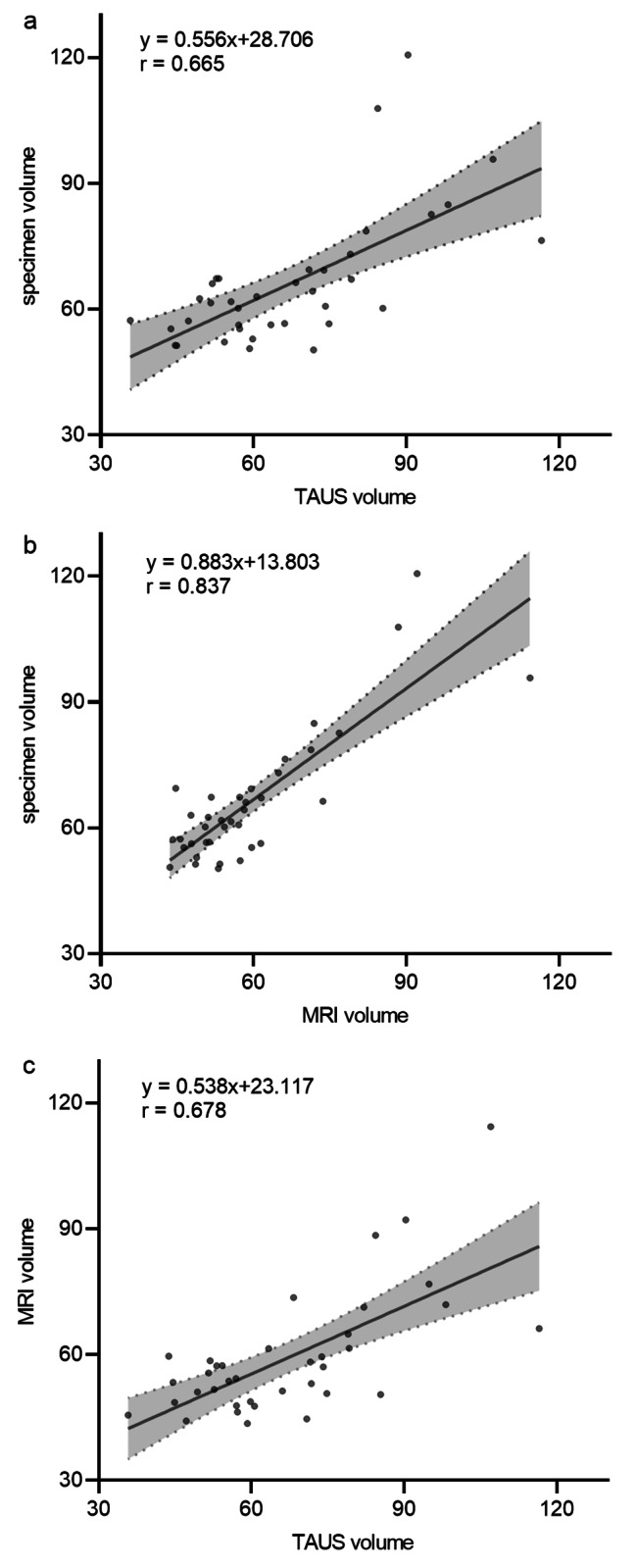
Scatterplot examination and linear regression analysis between different prostate volume measurements in patients with prostate volume bigger than 50 ml. (**a**) TAUS volume versus the specimen volume;(**b**) MRI volume versus the specimen volume; (**c**) TAUS volume versus MRI volume. MRI, magnetic resonance imaging; TAUS, transabdominal ultrasound

Patients in this cohort had PV on average less than 50 ml, so we further investigated the relationship between different measurements in patients with a volume greater than 50 ml (n = 37) (Fig. 5). In this subgroup, MRI volume was still correlated strongly with specimen volume (r = 0.837, p < 0.01), while TAUS volume showed only moderate correlation with specimen (r = 0.665, p < 0.01) or MRI volume (r = 0.678, p < 0.01). This suggested that MRI might be a more appropriate choice for measuring the large prostate.

## Discussion

Accurate measurement of PV was essential in the evaluation and management of prostate disease. For patients with benign prostatic hyperplasia, PV has been a powerful tool for the purpose of assessing symptom severity, predicting complications, and selecting appropriate treatments (medication or surgery) [10]. In terms of PCa, PV has been demonstrated to be effective in screening and risk stratification, especially when combined with PSA.

The best way to assess the accuracy of various volume measurements was to compare the results to the actual volume, which was equivalent to measuring the volume of the prostate specimen after RP. Formalin-fixed pathological specimens have been used for weighing instead of fresh surgical specimens in several studies [11, 12]. On the one hand, formalin fixation leads to tissue dehydration, which reduces the actual in vivo volume. On the other hand, pathological specimens may lead to an overestimation, as seminal vesicles and prostatic fat are usually not removed from the prostate. In this study, the volume of the specimen was measured immediately after RP using water displacement with removal of the periprostatic fat, seminal vesicles and vas deferens, thereby reducing the risk of under- or overestimation of the volume.

Historically, Planimetry-based assessment of PV was considered to be the closest to in vivo prostate size [13]. However, it was time-consuming, cumbersome, required special software, and was therefore not widely used in daily clinical practice. Although less accurate than Planimetry, ultrasonography using the ellipsoid formula has been widely used due to its speed, radiation-free nature, and cost-effectiveness. A systematic review showed a favorable correlation between the PV obtained by TRUS and surgical specimens, ranging from 0.70 to 0.90 [14]. In addition, we have also shown that PV measured by TRUS correlates well with TAUS, MRI, and specimen volume in our TRUS subgroup (Supplementary Table 1). However, TRUS was an invasive imaging modality that could cause discomfort and anxiety, particularly in patients with anal diseases such as hemorrhoid, anal fissure, and anal fistula. In fact, TAUS was typically the preferred choice for patients with lower urinary tract symptoms and was more commonly used to measure prostate dimensions. Furthermore, it was a non-invasive method that was well tolerated by the patient.

Previous studies showed a strong positive correlation and agreement between TAUS and TRUS, indicating that TAUS was an excellent surrogate for TRUS [15, 16]. However, few had explored the relationship between TAUS and fresh RP specimens and their relationship was not well defined. In the present study, TAUS overestimated specimen volume, but they were strongly associated and concordant when using the easily applicable ellipsoid formula. Varkarakis et al. also reported that TAUS overestimated the fresh specimen volume (4.61cm^3^), but the correlation was not reported [17]. Problems in measuring the longitudinal dimension have been suggested as a possible reason for the inaccuracy of the PV measured by TAUS, especially in larger prostates and when the bladder expansion was over or under full [18].

With the spread and improvement of MRI techniques, its higher spatial resolution, better soft-tissue contrast, and more complex computational capabilities made it superior in contouring the prostate, providing more precise and repeatable PV analysis. PI-RADS v2.1 aimed to standardize PV estimation and recommended routine reporting of PV based on MRI, by manual or automated segmentation or ellipsoid formula [9]. However, manual segmentation should be performed by an experienced radiologist or a trained non-radiologist and this approach was neither time-saving nor cost-effective [19]. ​Some types of automated segmentation have proven to be time efficient for accurate PV measurements, but require much more economy and generality [20]. It is worth mentioning that artificial intelligence is increasingly used in radiology, especially in prostate imaging. Deep learning-based prostate segmentation appears to be superior to traditional segmentation, and relevant studies have examined the feasibility of applying automated segmentation based on deep learning algorithm [21]. Nevertheless, the application of such models is mainly limited to academic research rather than clinical use.

Previous studies investigated the accuracy of the PV measured with the ellipsoid formula on MRI and discovered a high degree of association between the ellipsoid formula and the reference (manual planimetry or prostatectomy specimen). A prospective study included 21 patients who had undergone RP and found that PV measured on MRI using the ellipsoid formula had an excellent correlation coefficient with the volume of fresh RP specimen (r = 0.92) [22]. Bezinque et al. reported an excellent correlation between the PV calculated by the ellipsoid formula and MRI-R3D (manual segmentation by a radiologist) (ICC = 0.90), indicating that MRI using the ellipsoid formula provided an accurate measurement of PV [19]. In conclusion, PV estimation on MRI using the ellipsoid formula was a rapid technique with reasonable accuracy and reproducibility, and its general availability made it feasible for routine clinical use [23]. As in previous studies, our results demonstrated that the specimen volume for the entire cohort was highly associated with and underestimated by the volume measured by MRI [24, 25]. ​.

Several studies reported that the direction and magnitude of the difference was volume dependent. ​Matthews et al. compared PV measured by TRUS to specimen volume from 100 men diagnosed with PCa who underwent radical retropubic prostatectomy and reported that TRUS overestimated specimen volume for volumes less than 30cm^3^ and increasingly underestimated specimen volume for volumes greater than 30cm^3^ [26]. A similar study found that MRI appeared to overestimate and underestimate PV when the specimen volume was less than 35 cm^3^ and greater than 35 cm^3^, respectively [27]. However, this association was not found in TRUS in their study. Accordingly, the present study also explored whether the difference was volume-dependent. No statistically significant correlation was found between the difference and the specimen volume (P = 0.193) in the TAUS group. In the MRI group, we discovered that the direction and magnitude of the difference varied with specimen volume. In other words, if the specimen volume was < 39 ml, MRI overestimated the specimen volume; if the specimen volume was > 39 ml, MRI underestimated the specimen volume. In summary, MRI had a tendency to overestimate the smaller prostates but underestimate the larger ones.

Currently, many clinical risk-stratified prediction models and nomograms incorporate PV as a key predictor [28–30]. Although our data and previous studies demonstrated the superiority of MRI in measuring PV, capacity and resource limitations posed a challenge in delivering prebiopsy MRI for all men with suspected PCa. Therefore, we examined the relationship between TAUS-based and MRI-based PV and confirmed that they were highly associated and concordant, and the linear regression equation was established. ​However, the significance of such a conversion result needs to be further validated.

This retrospective study is not without limitations. First, the reproducibility of the volume measurements obtained could be limited by factors such as the inaccuracy of the inherent limitation of the ellipsoid formula used in this study, which assumed that the prostate had an ellipsoid-like shape that did not exist in fact. The shape of the prostate was highly variable and irregular, so any fixed formula that didn’t take shape into account was prone to error. Second, although all imaging tests were performed within 3 months prior to RP, the PV may have changed during this period due to tumor growth, which would affect the accuracy of the comparison between results. Third, the single-centric retrospective study design is another limitation of this study. Due to the limited sample size of this study, our findings need to be further verified in a well-designed, large-sample prospective study.

## Conclusions

The present study confirms a strong level of correlation and agreement between the specimen volume and the PV measured by TAUS and MRI, while MRI outperforms TAUS. In patients with a volume greater than 50 ml, MRI volume was still correlated strongly with specimen volume, while TAUS volume showed only moderate correlation with specimen or MRI volume. This suggested that MRI might be a more appropriate choice for measuring the large prostate.

## Electronic supplementary material

Below is the link to the electronic supplementary material.

**Supplementary Table 1** Correlation between prostate volume measured by TRUS, TAUS, MRI and specimen.

## Data Availability

The datasets generated and analyzed during the current study are available from the corresponding author on reasonable request.

## List of abbreviations

PV: Prostate volume
PCa: Prostate cancer
TAUS: Transabdominal ultrasound
MRI: Magnetic resonance imaging
ICC: Intraclass correlation coefficient
PSA: Prostate specific antigen
RP: Radical prostatectomy
CT: Computed tomography
