# Supplementary material for: Comparison of prostate volume measured by transabdominal ultrasound and MRI with the radical prostatectomy specimen volume: a retrospective observational study
Source: BMC Urol. 2023 Apr 17;23:62. doi: 10.1186/s12894-023-01234-5 (PMC10111778; doi:10.1186/s12894-023-01234-5)
Supplement: Supplementary file 1 — Supplementary Table 1 Correlation between prostate volume measured by TRUS, TAUS, MRI and specimen. [file 12894_2023_1234_MOESM1_ESM.docx]

**Comparison of Prostate Volume Measured by Transabdominal Ultrasound and MRI with the Radical Prostatectomy Specimen Volume: a retrospective observational study**

In our patient’s cohort, the number of patients with TRUS volume was 58 out of 106 because some patients had TRUS-guided biopsies performed at other facilities or some patients had no TRUS volume registered. In this subgroup, we found that PV on TRUS correlated well with PV on TAUS (r = 0.880, p<0.01), MRI (r = 0.889, p<0.01) and RP specimen (r = 0.853, p<0.01).

**Supplementary Table 1** Correlation between prostate volume measured by TRUS, TAUS, MRI and specimen.

| Variable | Pearson correlation coefficient | | | |
| --- | --- | --- | --- | --- |
|  | TRUS volume | Specimen volume | MRI volume | TAUS volume |
| TRUS volume | - | 0.853^*^ | 0.889^*^ | 0.880^*^ |
| Specimen volume | 0.853^*^ | - | 0.902^*^ | 0.850^*^ |
| MRI volume | 0.889^*^ | 0.902^*^ | - | 0.854^*^ |
| TAUS volume | 0.880^*^ | 0.850^*^ | 0.854^*^ | - |
| TRUS, Transrectal ultrasound; MRI, magnetic resonance imaging; TAUS, transabdominal ultrasound.  ^*^ p<0.01. | | | | |
